# Geometric Monitoring of Steel Structures Using Terrestrial Laser Scanning and Deep Learning

**DOI:** 10.3390/s26030831

**Published:** 2026-01-27

**Authors:** João Ventura, Jorge Magalhães, Tomás Jorge, Pedro Oliveira, Ricardo Santos, Rafael Cabral, Liliana Araújo, Rodrigo Falcão Moreira, Rosário Oliveira, Diogo Ribeiro

**Affiliations:** 1 iBuilt, School of Engineering, Polytechnic of Porto, 4249-015 Porto, Portugal; jorbm@isep.ipp.pt (J.M.); tosjo@isep.ipp.pt (T.J.); pmamo@isep.ipp.pt (P.O.); rps@isep.ipp.pt (R.S.); rac@isep.ipp.pt (R.C.); rem@isep.ipp.pt (R.F.M.); mro@isep.ipp.pt (R.O.); drr@isep.ipp.pt (D.R.); 2 CONSTRUCT, Faculty of Engineering, University of Porto, 4200-465 Porto, Portugal; 3 JF Metal, 4775-224 Barcelos, Portugal; 4 CIETI, School of Engineering, Polytechnic of Porto, 4249-015 Porto, Portugal

**Keywords:** structural geometric monitoring, EN 1090-2: 2020, terrestrial laser scanning, point cloud, YOLOv8, steel structures

## Abstract

Ensuring the quality and structural stability of industrial steel buildings requires precise geometric control during the execution stage, in accordance with assembly standards defined by EN 1090-2:2020. In this context, this work proposes a methodology that enables the automatic detection of geometric deviations by comparing the intended design with the actual as-built structure using a Terrestrial Laser Scanner. The integrated pipeline processes the 3D point cloud of the asset by projecting it into 2D images, on which a YOLOv8 segmentation model is trained to detect, classify and segment commercial steel cross-sections. Its application demonstrated improved identification and geometric representation of cross-sections, even in cases of incomplete or partially occluded geometries. To enhance generalisation, synthetic 3D data augmentation was applied, yielding promising results with segmentation metrics measured by mAp@50-95 reaching 70.20%. The methodology includes a systematic segmentation-based filtering step, followed by the computation of Oriented Bounding Boxes to quantify both positional and angular displacements. The effectiveness of the methodology was demonstrated in two field applications during the assembly of industrial steel structures. The results confirm the method’s effectiveness, achieving up to 94% of structural elements assessed in real assemblies, with 97% valid segmentations enabling reliable geometric verification under the standards.

## 1. Introduction

The Architecture, Engineering and Construction (AEC) sector has seen substantial advancements in the integration of digital technologies, including Building Information Modelling (BIM), Terrestrial Laser Scanning (TLS) and automated quality control tools. These innovations have transformed conventional tasks such as geometric quality control and structural inspection into faster, more reliable and increasingly automated digital workflows [1,2,3]. Despite this progress, many construction activities still rely on traditional visual inspections and selective manual measurements carried out by field inspectors, which may be inadequate for precise geometric assessments. A more systematic, data-driven approach involves using high-resolution spatial data captured through remote-sensing technologies. Among these, TLS is a static, close-range application of Light Detection and Ranging (LiDAR) principles, measuring distances by either the Time-of-Flight (ToF) measured by the delay between emitted and reflected pulses or by shifts in the laser wavelength [4]. This technique offers a cost-efficient approach to acquiring extensive geometric and visual data across the region of interest (ROI) [5].

In the current digitalisation era, laser scanners are extensively used for rapid and accurate object measurements through point cloud analysis. These point clouds capture the detailed geometry of an asset’s surface with high precision [6]. However, TLS data face several practical limitations. Oytun and Atasoy [7], using a Leica ScanStation P40, found that crack-width measurements are strongly influenced by material type, scanner-to-object range and angle of incidence. The authors concluded that cracks with widths below 2 mm could not be reliably measured regardless of scan resolution. Chacón, et al. [8] used TLS to capture “As-Built” point clouds of laboratory-scale stainless-steel frames subjected to vertical and lateral loads. These point clouds were converted into BIM-compatible wireframe models that preserved the measured deformations of known structural members for subsequent nonlinear analyses. However, stationary laser scanners are limited by line-of-sight constraints, leaving elevated or occluded areas unscanned. To overcome this, ref. [9] combined static TLS with a UAV-mounted Aerial Laser Scanner (ALS), which applies the same LiDAR technology but extends coverage through aerial surveying. In their circular-plan water-tower case study, TLS captured the lower sections, while the aerial survey covered the upper and occluded areas, albeit with lower precision. By slicing the merged point cloud into horizontal cross-sections and fitting ideal circles to each slice, the authors quantified radial deviations from the nominal geometry, reporting an average error of 13 mm. This hybrid approach combines the millimetric accuracy of TLS for ground-level surfaces with the broader coverage of ALS for areas beyond TLS reach.

Accurate alignment between TLS-acquired point clouds and digital models is essential for geometric assessment. When available, Ground Control Points (GCPs) provide the most rigorous spatial referencing across both laser scans and design models. However, in many cases, such as industrial construction sites, GCP deployment is either impractical or incomplete. In this context, local registration processes become crucial. Among them, the Iterative Closest Point (ICP) algorithm, introduced by [10], estimates rigid-body transformations by iteratively minimising distances between corresponding point pairs, though its accuracy depends on the initial alignment. To address this limitation, hybrid methods now combine global and local registration stages. For instance, ref. [11] developed an enhanced alignment pipeline integrating Sample Consensus Initial Alignment (SAC-IA) for coarse registration with a bidirectional KD-tree-optimised ICP (KD-ICP) for refinement. Other variants, such as the GF-ICP, integrate geometric features such as surface curvature and point density, reducing the dependence on an initial alignment [12].

Beyond measurement-oriented TLS applications, recent studies have explored geometry-driven approaches that interpret point cloud data at a structural level. In this context, ref. [13] proposed a framework for deriving simplified wireframe-based structural representation from point cloud data, focusing on the inference of structural elements to support condition assessment of civil infrastructures. While existing approaches primarily focus on reconstructing structural representations directly from point clouds, there remains a lack of automated, regulation-oriented methodologies specifically designed for geometric conformity verification.

Although point clouds are highly effective for detailed 3D modelling and spatial analysis, certain applications—especially those requiring faster processing or simpler data representation—benefit from 2D imagery. A key strategy involves projecting 3D point clouds onto 2D image planes. He, et al. [14] encoded colour and depth into standard 2D image formats, reducing data complexity without losing relevant geometry. Similarly, ref. [15] combined this projection technique with Deep Learning (DL) models for UAV-based bridge inspections, significantly improving crack detection by mitigating background interference and misclassification.

Recent studies have integrated DL algorithms into point cloud processing, enabling more automated and accurate workflows. In 2017, Qi et al. introduced PointNet [16], a pioneering DL model that directly processes unordered point sets. Subsequent architectures, like PointNet++ [17], OctNet [18], PointVGG [19] and PointGrid [20] incorporated hierarchical feature extraction and spatial partitioning, improving 3D object classification. Furthermore, SpiderCNN [21], PointCNN [22] and Dynamic Graph CNN (DGCNN) [23] extended these capabilities to segmentation and feature extraction, enhancing tasks such as point cloud segmentation, object detection, construction progress monitoring and structural integrity assessment.

In recent years, deep learning frameworks have been integrated into image-based object detection algorithms, relying on bounding-box representations to localise and classify features. Among these, Horizontal Bounding Boxes (HBBs) remain prevalent due to their simplicity and efficiency [24]. Modern detectors are typically divided into two-stage and single-stage approaches. Two-stage networks, such as Faster R-CNN, first generate region proposals using a Region Proposal Network (RPN) before refinement and classification. Fu, et al. [25] embedded the StairNet module within a Faster R-CNN architecture to detect pavement cracks, outperforming conventional CNNs, including GoogLeNet [26], VGG16 [27], ResNet34 [28] and MobileNet [29,30]. Single-stage detectors, in contrast, merge region proposal and classification into a unique CNN step. The You Only Look Once (YOLO) series exemplifies this, achieving real-time detection, precise localisation and class prediction [31]. The YOLO process comprises the following: (i) grid division of the input image, with each cell responsible for predicting object localisation (bounding box) and confidence scores (class probabilities); (ii) bounding box and class prediction; and (iii) final detection using class probabilities per grid cell [32,33]. Successive versions—up to YOLOv13—have progressively enhanced accuracy and performance [34].

Some applications in the civil engineering sector have demonstrated impressive performance when applying YOLO to multiple computer vision tasks [29,30,35,36]. In the domain of semantic image segmentation, YOLOv5 and YOLOv8, developed by Ultralytics, represent two of the most widely utilised approaches. Casas, et al. [37] conducted a comparative study assessing their respective efficiency, robustness and adaptability for complex engineering tasks. The researchers found YOLOv8 outperformed YOLOv5 in terms of speed, accuracy and generalisation capabilities. Kumar, et al. [38] employed an earlier version, YOLOv3, for real-time detection of cracks and spalling on concrete structures. This methodology used HBBs to localise damage, successfully detecting cracks wider than 0.20 mm from 400 mm away. Similarly, ref. [39] used YOLOv5 to develop a scalable tool for bridge maintenance and condition monitoring, capable of detecting multiple defects in reinforced concrete bridges. Additionally, ref. [40] applied YOLOv5 to detect and classify efflorescence on brick facades, enabling automatic differentiation between bricks that require simple cleaning and those requiring structural repair.

However, HBBs cannot always accurately represent objects with arbitrary orientation. To address these limitations, researchers developed advanced mathematical approaches for representing Oriented Bounding Boxes (OBBs), which can rotate to precisely match the orientation of an object. In contrast to HBBs, OBBs offer the flexibility to align the bounding box with the object’s orientation, resulting in a more accurate and compact representation of its actual shape [41,42,43]. This approach has enabled various applications in fields where objects may appear at arbitrary orientations, such as remote sensing for aerial and satellite imagery [41,42,44], text detection [45] and 3D object detection [46,47]. Nonetheless, these methods adopt a five-parameter representation—consisting of the object’s centre coordinates, width, height and rotation angle—which can lead to angular discontinuities and parameter inconsistencies. Qian, et al. [48] addressed the OBB challenges by shifting from the common five-parameter labels to an eight-parameter corner-point regression model that directly predicts the four vertex coordinates of the OBB. An alternative approach is to frame OBB detection as a segmentation task rather than a direct coordinate regression. For instance, ref. [42] introduced the Mask-OBB, a semantic-attention mask representation that transforms OBB estimation into a pixel-level classification problem. The methodology uses predicted masks to generate precise OBBs, thereby effectively avoiding the boundary ambiguity and discontinuity problems. Conversely, ref. [44] developed a methodology specifically designed for high-resolution remote sensing imagery, aiming to address the challenges of segmenting complex object boundaries.

This paper presents a methodology that integrates TLS and DL techniques to automate the geometric control of industrial steel structures during assembly. The proposed approach addresses limitations of conventional manual inspection and heuristic-based procedures through the following key contributions:

Use of terrestrial laser scanning and deep learning to replace traditional geometric control methods that rely on manual, error-prone measurements.

Automation of wireframe extraction from IFC models, enabling precise alignment with point cloud data.

YOLOv8-based instance segmentation of 2D steel cross-sectional projections, enabling reliable identification of commercial steel profiles even under noise, occlusion and incomplete geometry conditions [49].

A synthetic 3D data augmentation strategy to generate projection datasets for YOLOv8 training, representative of real-world conditions.

Comprehensive field validation following EN 1090-2:2020 [50] criteria for geometric assembly control of industrial steel structures.

## 2. Methodology

**Figure 1:**
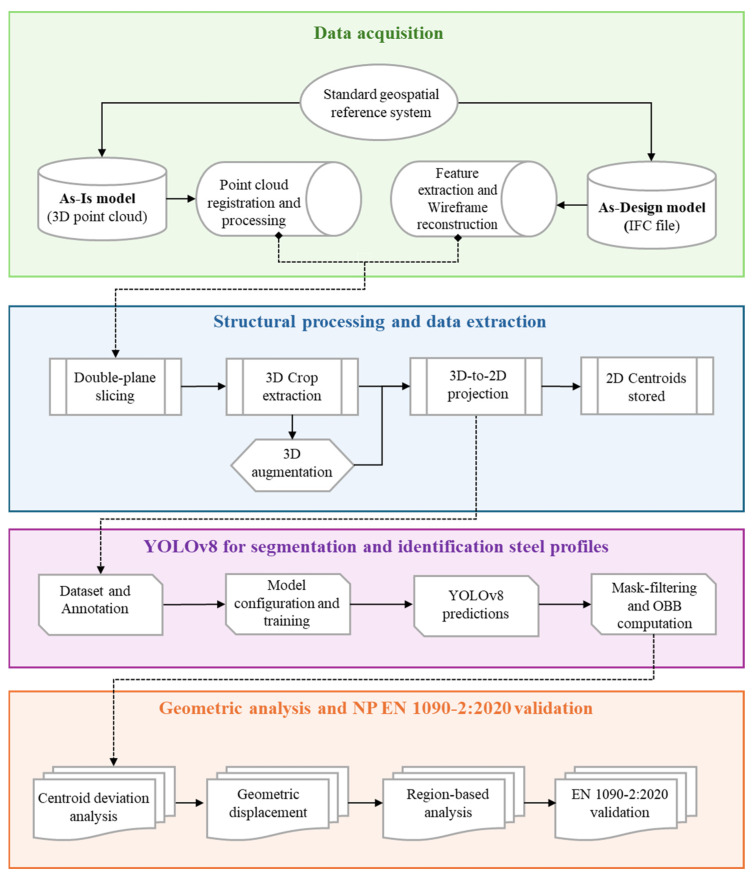
Overview of the proposed methodology.

This section provides an overview of the proposed framework for the automated geometric control of industrial steel structures. The framework integrates high-resolution 3D point cloud data, captured using a TLS, and structural information from IFC models to enable accurate comparisons between design and actual construction. Additionally, for improved automation and accuracy, a custom YOLOv8 segmentation model was trained to detect and classify cross-sectional steel profiles from 2D projections of the 3D point cloud data. The YOLOv8 model enabled robust feature extraction for the automatic assessment of geometric deviations according to EN 1090-2:2020. The methodology comprises four main stages: (i) data acquisition, (ii) structural processing and data extraction, (iii) YOLOv8 segmentation and identification of steel profiles, and (iv) geometric analysis and EN 1090-2:2020 validation. Figure 1 provides an overview of the complete methodology.

### 2.1. Data Acquisition

Accurate geometric control begins with reliable access to two essential data sources: the 3D point cloud model, which reflects the current physical state of the structure, and the IFC model, which contains the intended design configuration. These models enable direct geometric comparison between the as-built and as-designed conditions. To support this, both datasets are aligned within a shared geospatial reference system, forming a reliable foundation for subsequent geometric deviation analysis.

In order to ensure the highest standards regarding data quality and acquisition conditions, the TLS equipment and survey settings used to capture the As-Is model are described in the following subsection.

#### 2.1.1. Equipment and Acquisition Settings

The As-Is structural configuration was geometrically captured using terrestrial laser scanning, relying on commercial laser scanning systems that are suitable for construction-scale geometric monitoring and quality control. The methodology was tested with two laser scanners: the Leica BLK360 G1 and the Leica RTC360 (Leica Geosystems, Heerbrugg, Switzerland). Table 1 summarises the main technical characteristics of the two TLS. Although both scanners are based on ToF ranging and provide full panoramic coverage, their technical characteristics differ in terms of ranging accuracy, acquisition speed and operating range.

**Figure 2:**
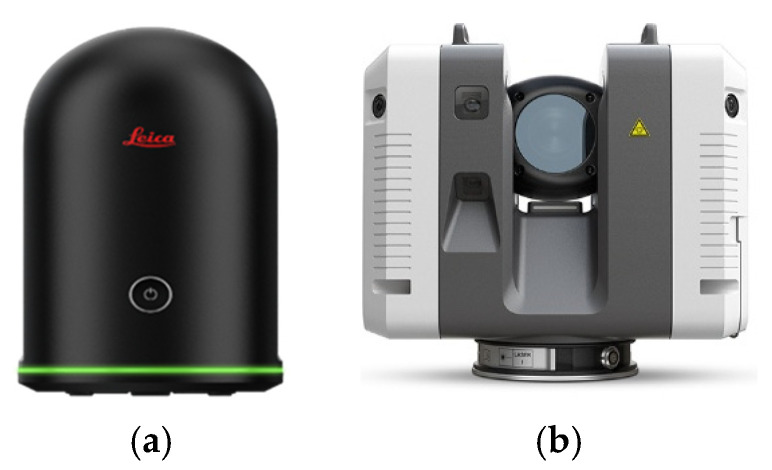
Terrestrial laser scanners employed: (**a**) Leica BLK360 G1; (**b**) Leica RTC360 (adapted from [51]).

The Leica BLK360 G1 (Figure 2a), characterised by its low weight and compact form factor, offers a nominal ranging accuracy of ±4 mm at 10 m and an effective maximum range of approximately 60 m. Under these specifications, the scanner produces point cloud data of sufficient geometric fidelity to support the subsequent processing stages.

The Leica RTC360 (Figure 2b) provides an enhanced ranging performance, combining nominal ranging accuracy of ±1 mm at 10 m with an effective maximum range of 130 m and high measurement rate. A distinguishing feature of this system is the integration of a Visual-Inertial System (VIS), which uses video-based motion tracking with inertial measurements to support real-time, targetless registration between consecutive scan positions. This potentiality improves registration stability and reduces cumulative alignment errors during large surveys.

**Figure 3:**
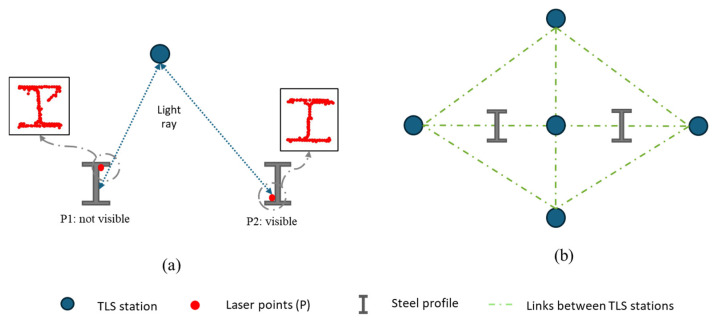
Influence of TLS scan positioning on cross-sectional data quality: (**a**) non-optimal scanner position; (**b**) optimised scanning configuration ensuring maximum visibility and accurate acquisition.

The accuracy of the data fundamentally depends on careful pre-survey planning and scanner configuration. This includes determining the optimal number of scans and strategically positioning the scanner to ensure full visibility of the steel element cross-sections. Due to the complex geometry of steel profiles, inappropriate scan positioning may result in occlusions and incomplete sampling of critical geometric features. Figure 3 represent the influence of scan positioning on data quality. Figure 3a highlights a non-optimal acquisition scenario, where the interaction between the TLS light ray and the geometry of the steel profile leads to partial or distorted cross-sectional information. In contrast, Figure 3b presents the proposed scanning strategy to ensure full visibility and accurate capture of the complete geometric configuration of the steel element.

#### 2.1.2. As-Is Model

The As-Is 3D model is generated from a TLS survey using medium-density scanner settings. An iPad equipped with a LiDAR sensor captures additional detail in hard-to-reach areas, and real-time pre-alignment is performed in Leica Cyclone FIELD 360.

For post-survey processing, the raw scans are imported into Leica Cyclone REGISTER 360 PLUS for global registration and alignment, minimising linking errors. The registered point cloud is georeferenced using on-site GCPs obtained from a topographic survey to ensure consistency within the shared geospatial system. Irrelevant components are then removed, retaining only the structural elements. The dataset is optimised through radius-based outlier removal and voxel down-sampling to reduce noise and density while preserving geometric features.

#### 2.1.3. As-Design Model

**Figure 4:**
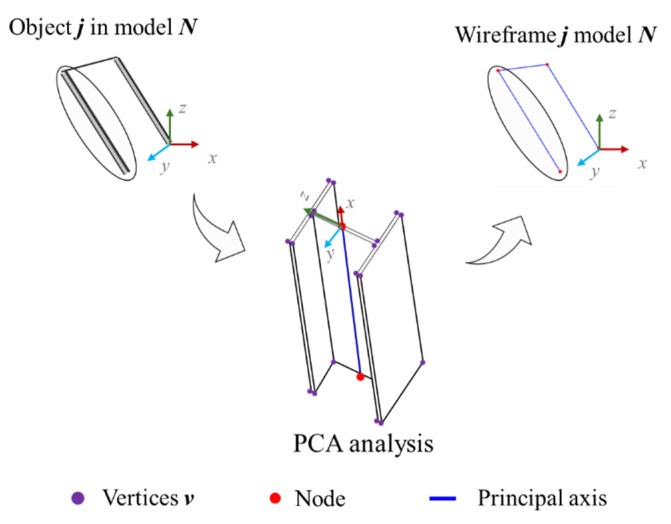
PCA application for wireframe extraction.

The As-Design data analysis process begins with the extraction of geometric and structural features from the IFC file describing the BIM asset. An algorithm processes the IFC data, extracting element information, including columns and beams, calculating precise endpoints, node coordinates, lengths and related metadata. Principal Component Analysis (PCA) [52] is then applied to determine the principal axis of each object j in the model N and compute its 3D node coordinates, as shown in Figure 4. Vertex coordinates v are arranged in a matrix X(v×m), containing each vertex’s normalised 3D spatial coordinates after mean subtraction. The covariance matrix $C_{x}$ is calculated to reveal the object orientation, as in (1).

(1) $$C_{x}=\frac{1}{n-1}X^{T}X$$

This matrix is decomposed into eigenvectors and eigenvalues using $C_{x}\cdot P=P\cdot \Lambda$, where P defines the principal-axis directions and Λ represents the variance of each principal component (PC). Geometrically, the transformed data matrix T=X·P (score matrix) projects the original data onto PCs, from which node coordinates are derived to reconstruct the wireframe of elements j.

### 2.2. Structural Processing and Data Extraction

**Figure 5:**
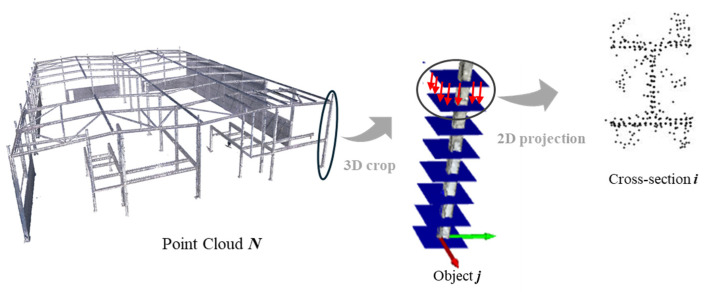
Procedure of projecting 3D point cloud crops onto 2D slicing plane surfaces to generate steel cross-sectional images.

Following the data acquisition phase, both data sources—the As-Is point cloud and the As-Designed wireframe—are processed together in an integrated workflow. Operating within a shared geospatial reference system, the wireframe provides the geometric centrelines of structural elements, which are used to slice the As-Is point cloud into multiple parallel double planes oriented perpendicularly to each element axis. The resulting 3D point cloud segments, located between each double-plane pair, are then projected into 2D cross-sectional images for each structural element. This process is repeated along each element axis, enabling data extraction at multiple key positions. The overall procedure is schematically illustrated in Figure 5. Each generated image is stored with a standardised resolution and a fixed metric scale for consistency across all sections, following a structured naming convention including element ID, section type and projection index. In the adopted configuration, the projection window corresponds to a 1 m × 1 m area, with a constant image resolution, resulting in a known pixel-to-metric relationship (1 pixel ≈ 1 mm). This enables subsequent processing and comparison to be performed in true scale, independent of visual representation. The proposed workflow remains scalable for both real and synthetic point clouds, provided a shared geospatial reference system is maintained.

**Figure 6:**
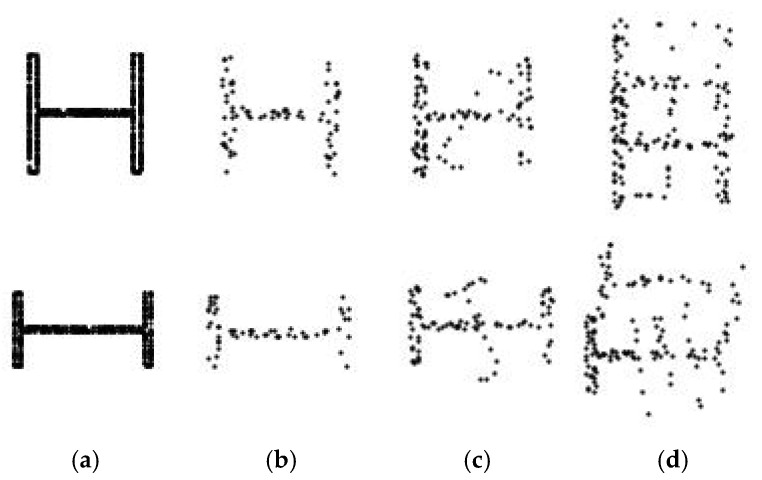
Example of the 3D data augmentation process applied to a synthetic HEA and IPE cross-sections: (**a**) clean projection of the original synthetic segment, (**b**) Gaussian noise and dropout augmentation, (**c**) synthetic profile insertion, (**d**) simulated cross-section duplication.

At this point, it was observed that real-world cross-sectional images frequently contain noise, occlusions and incomplete regions which hinder accurate analysis and model training. To mitigate this limitation, a 3D augmentation process based on synthetic data was implemented by applying controlled perturbations to extracted 3D segments prior to projection. The structured naming convention ensures consistent application of several 3D augmentation techniques: (i) Gaussian noise injection; (ii) voxel down-sampling at multiple scales; (iii) directional point dropouts to simulate occlusions or missing points; (iv) synthetic profile insertion with standardised I-section profiles such as HEA, HEB and IPE; and (v) simulated ghost points to replicate reflection or duplication effects. The results of these augmentations are shown in Figure 6, illustrating the progression from a clean synthetic projection to increasingly augmented variations in the same cross-section class. This enriched and varied dataset of labelled 2D cross-sectional images enabled robust deep learning model training for steel cross-section segmentation and classification, as detailed in Section 2.3.

### 2.3. YOLOv8 for Segmentation and Identification of Steel Profiles

In order to enhance the automation and reliability of cross-sectional analysis, a YOLOv8 instance segmentation model was trained to detect, classify and segment steel profiles’ cross-sections in 2D image projections. It should be noted that the proposed methodology does not aim to directly measure profile sectional properties from raw point cloud data. Instead, steel cross-sections are identified and classified using a deep learning model, and the corresponding geometric properties are assumed based on standardised commercial profile definitions.

Recognising structural profiles from real-world data, which often contain noise and partial occlusions, requires a robust, high-performance vision model capable of delivering fast and accurate predictions. For this reason, YOLOv8 was selected over other alternatives such as YOLOv5 and Mask R-CNN, as it offers an optimal balance of accuracy, inference speed and ease of deployment. In contrast to Mask R-CNN, a two-stage model with high segmentation accuracy but also high computational cost, YOLOv8 functions as a one-stage, anchor-free detector with an integrated segmentation head based on a prototype mask. Its architecture integrates a multi-scale backbone and optimised head, ensuring fast and precise segmentation. YOLOv8 was chosen to align with the proposed methodology, designed for standard-hardware deployment and scalable analysis during structural assembly.

**Figure 7:**
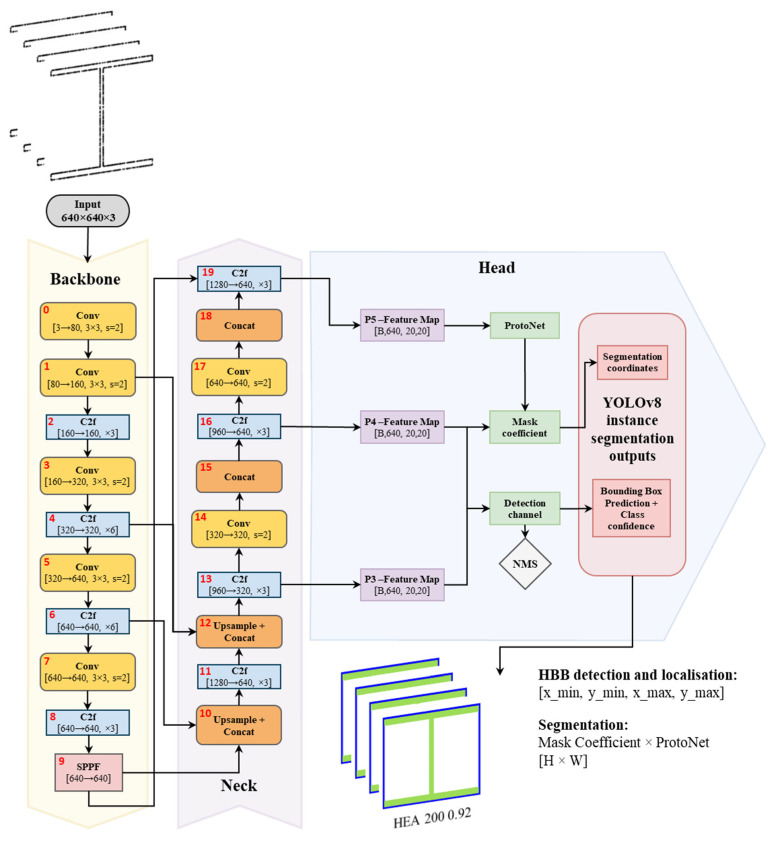
YOLOv8 segmentation pipeline.

An integrated overview of the YOLOv8 segmentation architecture is presented in Figure 7, illustrating the complete processing pipeline from raw 2D cross-sectional inputs to final instance-segmentation outputs, including class labels, HBBs and segmentation masks. This approach enables direct recognition of structural elements despite partial occlusions inherent to digital acquisition, reducing dependence on manual profile identification or heuristic reconstruction.

#### 2.3.1. Dataset and Annotation

The YOLOv8 model was trained on a custom dataset combining both augmented synthetic projection images and real data acquired through LiDAR-based surveys. The dataset includes 100 distinct classes representing structural steel cross-sections from the HEA, HEB and IPE series, each considered in two orientations: a standard configuration and a rotated variant, labelled with the suffix “R” (e.g., HEA100 and HEA100R). This range covers profiles ranging from 100 to 500 mm in height in each family, enabling the model to recognise and classify the full spectrum of commonly used steel sections. Orientation-based labelling follows engineering practice, as rotation influences structural behaviour and enables the validation of correct placement during assembly.

For rigorous geometric analysis, a systematic data annotation procedure was established. Images were first organised by class, each beginning with a clean synthetic image to ensure accurate initial labelling. Roboflow [53] enabled annotation duplication across images, ensuring consistent labelling of synthetic and real data and preserving geometric consistency. This systematic annotation process resulted in a robust model capable of recognising commercial steel cross-sections under multiple geometric and orientation conditions.

#### 2.3.2. Training Strategy and Prediction Results

The training process aimed to maximise the model’s generalisation and robustness through a mix of clean and augmented synthetic images, supplemented by real data from several field campaigns. Optuna [54] was used for hyperparameter tuning, and training employed the AdamW optimiser, which adjusts learning rates and applies weight decay to stabilise gradient updates and reduce overfitting. Initially trained solely on synthetic data, the model showed strong performance on low-noise real images but reduced accuracy on complex cases, revealing the need for fine-tuning. A second training cycle incorporated real projections and additional synthetic augmentation while retaining previously learned features. Table 2 summarises the main training hyperparameters, including device configuration, dataset split, image counts, resolution, batch size, epochs and optimiser settings.

**Figure 8:**
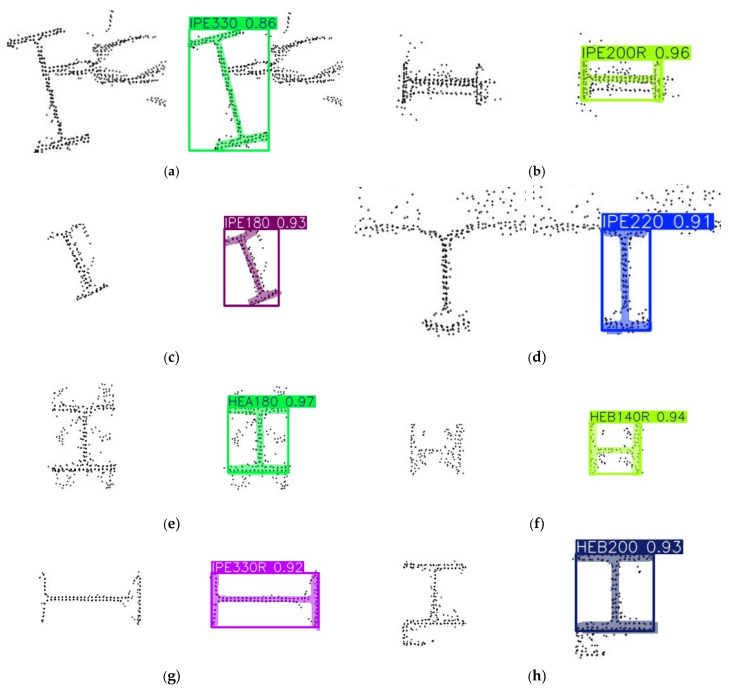
YOLOv8 segmentation results on real 2D cross-sectional images with confidence levels: (**a**) IPE330 (0.86), (**b**) IPE200R (0.96), (**c**) IPE180 (0.93, rotated), (**d**) IPE220 (0.91, noisy), (**e**) HEA180 (0.97, base cut revealing the bolt connections), (**f**) HEB140R (0.94), (**g**) IPE330R (0.92), (**h**) HEB200 (0.93, ignoring the L-section).

The model’s predictions include both the profile class identification and object segmentation, enabling localisation and geometric analysis on previously unseen images. Its ability to generalise to real data stems from the diverse and extensive augmentation strategy. Furthermore, YOLO’s output predictions systematically store geometric data for each detection, including HBB coordinates that define the axis-aligned rectangular region enclosing the object, and segmentation mask coordinates which provide a pixel-level delineation of its shape and position. Table 3 presents the main validation metrics obtained from the final training configuration, including bounding box (B) and segmentation mask (M) performance, showing consistently high precision (P), recall (R) and mAP scores across both tasks. Figure 8 illustrates several examples of the model’s predictions applied to real-world cross-sectional projections.

#### 2.3.3. Prediction-Based Filtering and OBB Computation

Accurate geometric analysis of point cloud projections requires post-processing computer vision methods to isolate key features and improve data quality. Here, segmentation-driven filtering is integrated with OBB computation for precise characterisation. Each segmentation mask generated by the model is overlaid on the corresponding image to isolate the object. A binary mask assigns a value of 1 to the object and 0 to the background, effectively removing noise. This processed output forms the basis for accurate downstream geometric analysis.

**Figure 9:**
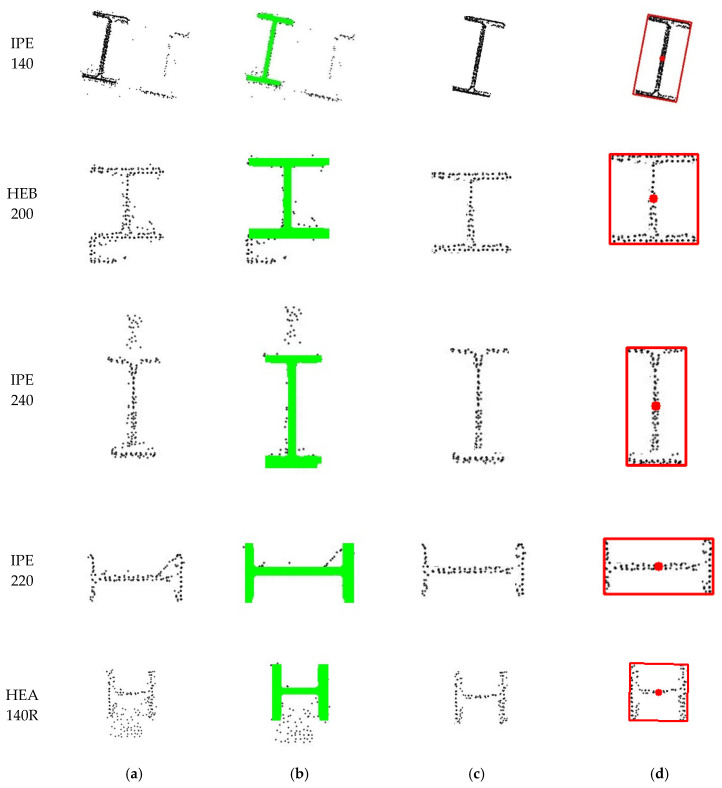
Step-by-step visualisation of the segmentation and geometric analysis pipeline: (**a**) original raw 2D cross-sectional image, (**b**) segmentation mask generated by the YOLOv8, (**c**) filtered image with the isolated section, (**d**) computed OBB, with red dot representing the OBB centroid.

The resulting filtered image is processed using the OpenCV library [55] to extract geometric boundaries of the segmented object. Specifically, the *findContours* function detects the object’s outer edges and generates detailed contour representations. These contours are then processed with the *minAreaRect* function to compute the minimum-area rectangle enclosing the object, yielding an OBB that compactly represents the profile’s shape and rotation. The entire pipeline is exemplified in Figure 9, which illustrates each processing stage.

### 2.4. Geometric Analysis and EN 1090-2:2020 Validation

Geometric quality control under EN 1090-2:2020 is assessed by measuring deviations between the executed structure (As-Is model) and the design intent (As-Design model). The proposed methodology validates steel structural elements against five key geometric deviation criteria defined in the standard, encompassing planimetric positioning, column inclination and straightness and beam inclination and straightness. These criteria were selected for their structural relevance and ability to represent overall geometric conformity; however, EN 1090:2-2020 defines additional tolerances for other assembly conditions not addressed in this study. The applicable deviation for each criterion depends on the execution class and element length (l) or height (h). Table 4 summarises the corresponding tolerance limits and geometric scenarios defined in EN 1090-2:2020.

The proposed methodology employs a centroid-based analysis of the computed OBB, extracted from the segmented-filtered images. Two comparison strategies are implemented, depending on the specific normative criterion considered.

(a) Internal comparison within local element—This strategy evaluates consistency along a single element rather than its absolute position in the global structure. Centroids from different cross-sectional slices of the same element are compared without reference to the As-Design model. It applies to criteria C2, C3, C4 and C5.
(b) Direct comparison with the As-Design model—This strategy involves comparing each OBB centroid at the bottom to its corresponding reference point in the As-Design wireframe. It is specifically used to assess planimetric positioning accuracy (criterion C1).

**Figure 10:**
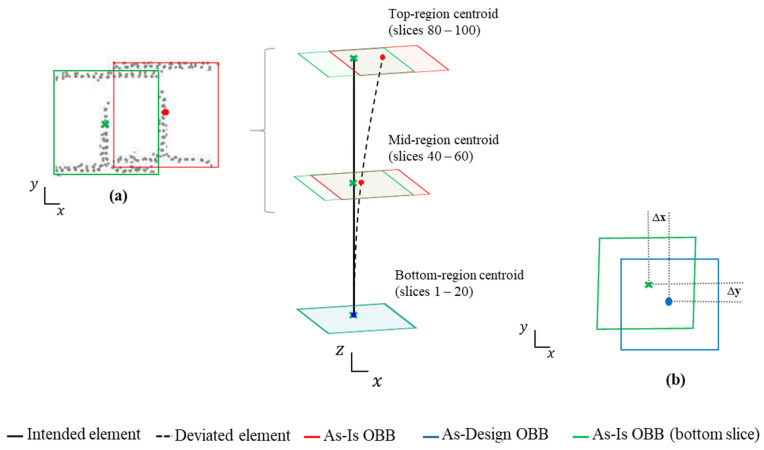
Geometric measurement strategies based on OBB centroid: (**a**) internal comparison within local element, (**b**) direct comparison with the As-Design model.

The analysis measures both translation displacements (Δx, Δy) between the As-Design reference centroid and the As-Is centroids. The validation process focuses on three distinct regions of each element: the Bottom (slices 1–20), the Mid (slices 40–60) and the Top (slices 80–100). These intervals cover structurally relevant sections and ensure spatial coverage and methodological robustness, even when data are incomplete or partially predicted. Figure 10 illustrates the two centroid-based measurement strategies defined for geometric validation following EN 1090-2:2020. Each criterion is assessed within predefined slice ranges corresponding to its geometric purpose: C1 uses strategy (b) on the bottom-region relative to its As-Design reference; C2-C5 use strategy (a), where C2 compares bottom and top centroids; C3 evaluates mid-span deviations along the element axis; C4 analyses left- and right-side elevations; and C5 examines mid-span within the element axis. The effectiveness of this strategy depends on accurate alignment between the As-Is and As-Design models.

**Figure 11:**
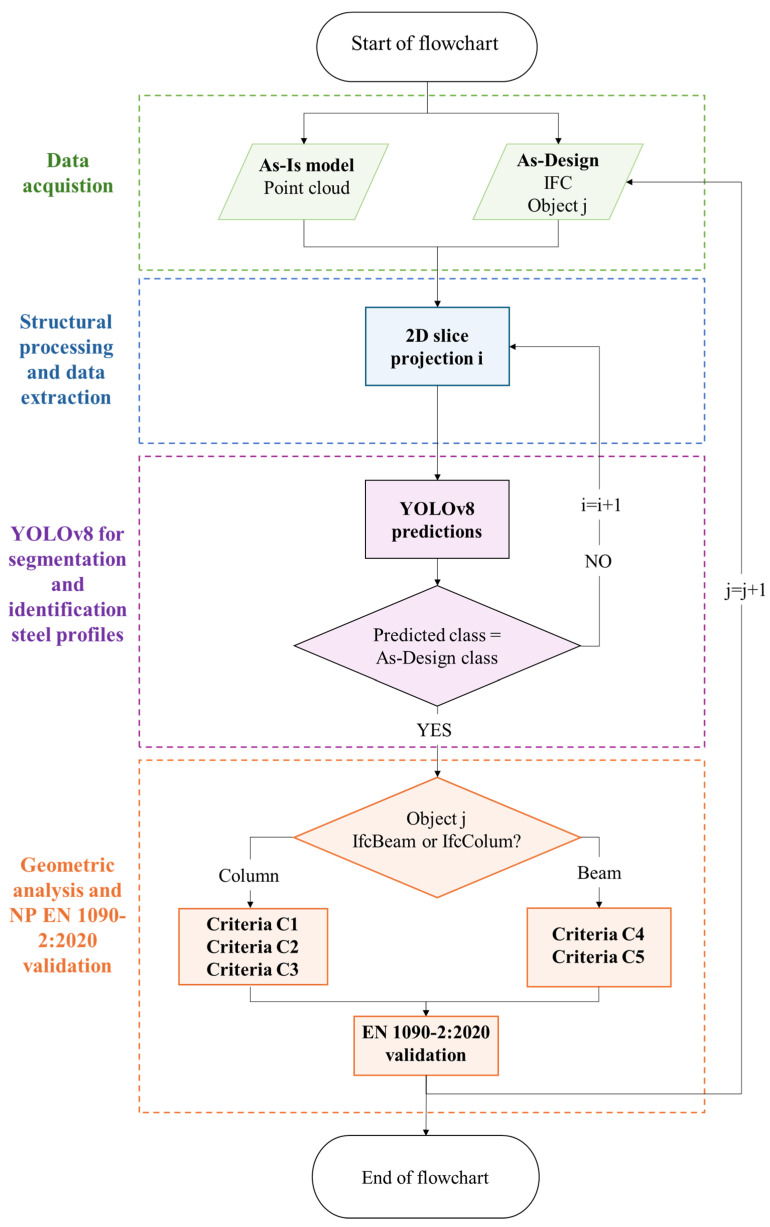
Flowchart of the proposed methodology.

Figure 11 summarises the proposed automated workflow for geometric control of industrial steel structures. The process integrates multiple data sources and analytical modules into a continuous pipeline that links acquisition, processing, recognition and validation. The As-Is point cloud model and the As-Design information are jointly processed to generate a standardised 2D projection, enabling the YOLOv8 segmentation model to detect and classify structural cross-sections automatically. Rather than relying on direct estimation of sectional dimensions from point cloud geometry, the workflow establishes cross-sectional shapes through YOLOv8-based classification of standardised steel profiles.

The integration of AI-based recognition with rule-based geometric validation enables each element to be assessed according to its typology (IfcColumn or IfcBeam) and verified against the applicable EN 1090-2:2020 tolerance criteria. This workflow consolidates all intermediate steps—from data alignment to deviation quantification—into a single, automated framework for consistent quality control.

## 3. Application and Discussion

This section presents the results derived from applying the proposed geometric control methodology to two real-world case studies. The analysis is focused on verifying their geometric compliance based on the EN 1090-2:2020.

### 3.1. Case Studies

**Figure 12:**
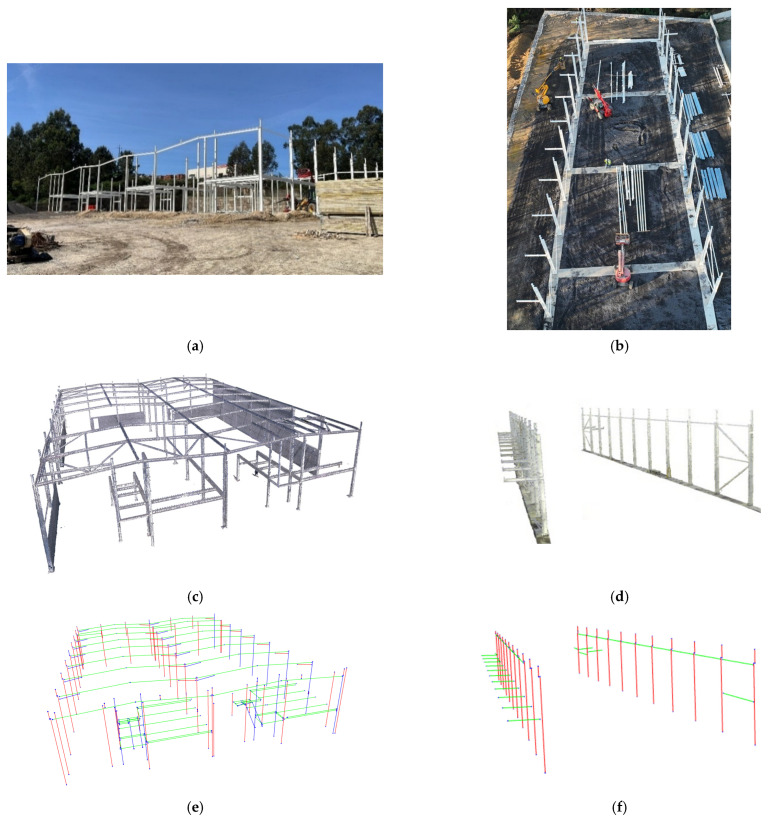

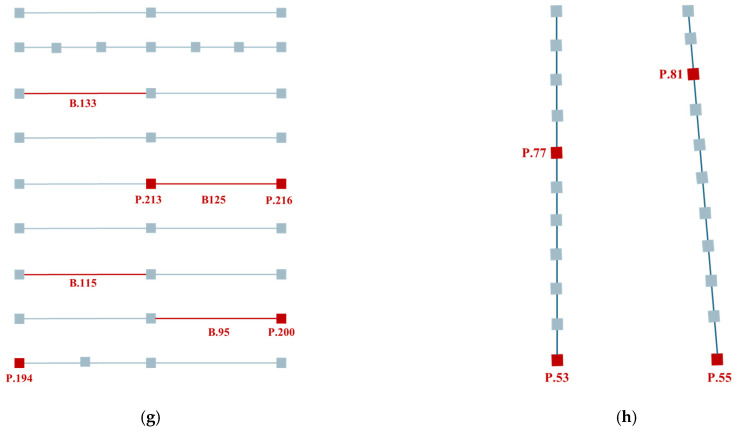
Survey data and model representations: (**a**) Case I—Amarante, (**b**) Case II—Maia, (**c**) Case I—captured 3D point cloud, (**d**) Case II—captured 3D point cloud, (**e**) Case I—As-Design wireframe reconstruction, (**f**) Case II—As-Design wireframe recontruction, (**g**) Case I—structural elements validated under EN 1090-2:2020, with analysed elements highlighted in red, (**h**) Case II—structural elements validated under EN 1090-2:2020, with analysed elements highlighted in red.

The methodology was tested on two industrial steel assembly case studies located in Amarante and Maia (Porto district, Portugal), designated as Case I and Case II, respectively. Figure 12a,b illustrates the structural assembly stages during the surveys. Case I, evaluated at a more advanced execution phase, had all columns and most beams installed, supporting a comprehensive element-based validation. Case II, captured at an earlier phase of construction, with only columns and partial bracings, presented greater geometric complexity due to its inclined façades.

Both surveys used TLS to obtain high-resolution 3D point clouds of the structure. Two different pieces of equipment were used: Leica BLK360 G1 for Case I and Leica RTC360 for Case II. Real-time pre-alignment in Cyclone FIELD 360 and subsequent bundle adjustment in Cyclone REGISTER 360 PLUS minimised registration errors. The processed point clouds are shown in Figure 12c,d.

Referential theoretical geometries were derived from As-Designed IFC models through the application of a PCA-based wireframe extraction algorithm. Figure 12e,f depicts the resulting wireframe reconstruction models for both cases. Georeferencing required two different approaches: for Case I, lacking georeferentiation, alignment between the As-Is and As-Designed models was achieved with an ICP-based registration, yielding mean and median residual errors of 2.0 cm and 1.3 cm, respectively; for Case II, a georeferenced IFC was provided and on-site GCPs were deployed, allowing direct CRS alignment and a more accurate geometric comparison. Figure 12g,h highlights the structural elements assessed under EN 1090-2:2020. These were validated according to the five geometric-deviation criteria listed in Table 4.

### 3.2. Geometric Control

#### 3.2.1. Case I—Industrial Building in Amarante

The geometric validation for Case I focused on representative columns and beams on two of the four identical modular halls, ensuring spatial coverage and structural representativeness. Partition walls, placed between some columns, restricted access to certain elements, reducing the analysis to the ones fully captured. The quantitative results are summarised in Table 5, which consolidates the measured deviation of the selected elements under the five geometric criteria defined in EN 1090-2:2020.

**Figure 13:**
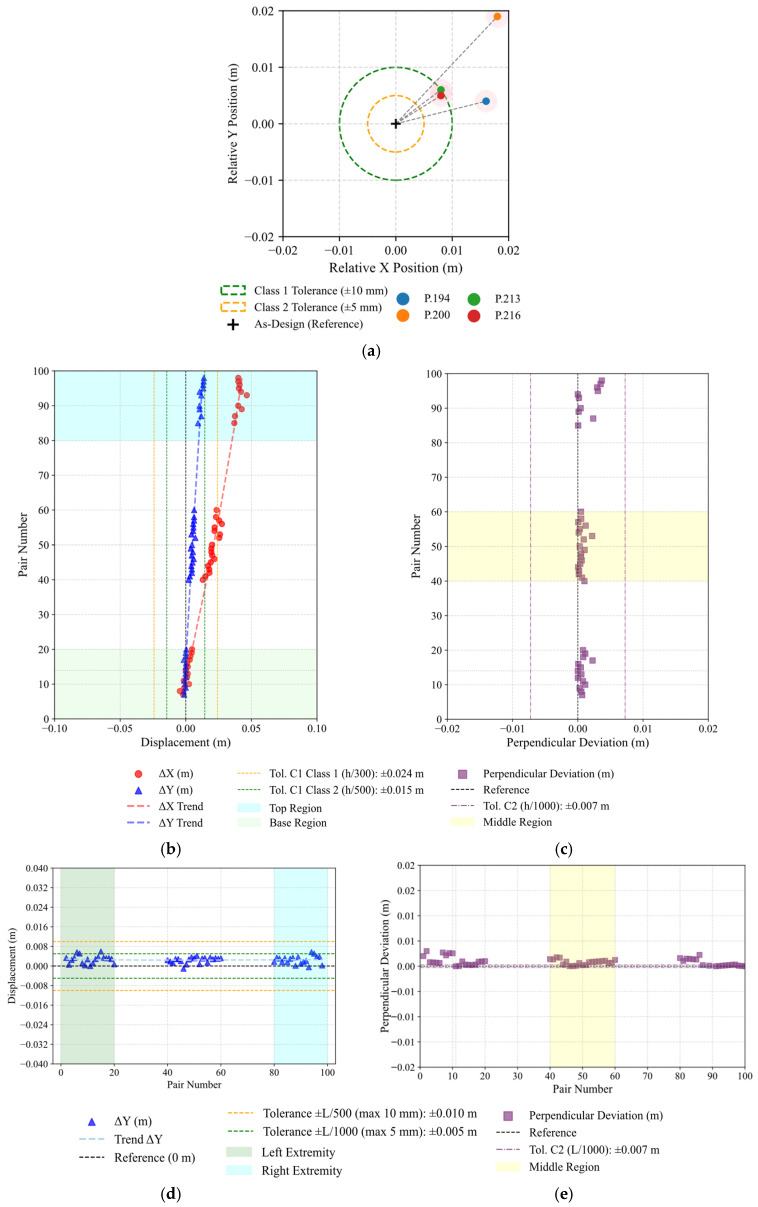
Visual representation of geometric deviation assessment for Case I—Amarante: (**a**) Criterion C1—Planimetric positioning of columns, (**b**) Criterion C2—column inclination, (**c**) Criterion C3—column straightness, (**d**) Criterion C4—beam vertical inclination, (**e**) Criterion C5—beam mid-span straightness.

Planimetric positioning of the columns at their base was evaluated under Criterion C1. Elements, such as P.213 and P.216, remain within the ±1 cm Class 1 tolerance. Columns P.194 and P.200 exhibit slightly larger offsets along both directions, consistent with minor residual deviations from the ICP-based alignment. The schematic distribution and magnitude of these deviations are shown in Figure 13a.

Criterion C2 evaluates column inclination by measuring horizontal displacements between base and top centroid regions. The evaluation considered both X and Y directions to verify uniform vertical alignment across planimetric axes. For 7.35 m high columns, the Class 1 tolerance of ±2.4 cm was adopted. Most results exceed the admissible tolerances; nevertheless, the analysis effectively identified and quantified this inclination behaviour, as illustrated in Figure 13b, where the blue and green highlighted zones correspond to the top and base regions of the column, respectively.

The straightness criterion quantifies the maximum perpendicular distance (Δm) of the mid-region from the line connecting the base and top centroids. All tested columns remained below the ≈0.7 cm tolerance limit, confirming the high sensitivity to small displacements, capable of detecting variations in the millimetre range, as evidenced in Figure 13c, where the middle zone is represented in yellow.

Criteria C4 and C5 extend the geometric evaluation to beam elements, addressing vertical inclination and mid-span straightness, respectively. The assessment of vertical inclination considered the end-to-end height difference (Δy) between beam extremities, limited by the Class 1 tolerance of ±l/500 and limited to a maximum of 1 cm, for the 7.35 m member. Mid-span straightness was analysed through the maximum perpendicular deviation (Δm) of the central region from the line connecting both ends, with an admissible limit of ±1.5 cm. As shown in Table 5 and Figure 13d,e, most beams satisfy these requirements. Minor exceedances in B.125 and B.133 indicated local execution deviations, while the remaining element display sub-centimetric accuracy. The reliable detection of these aerial elements confirms the suitability of TLS surveys for precise beam-level validation, indicating the methods adaptability.

Overall, Case I demonstrates satisfactory compliance with EN 1090-2:2020. The methodology consistently delivered accurate interpretation of the results across all evaluated criteria, proving effective even under partial visibility and non-georeferenced conditions.

#### 3.2.2. Case II—Industrial Building in Maia

The survey for Case II was conducted at an earlier assembly stage, with only columns and partial bracing installed. During acquisition, on-going cross-bracing installation caused slight dynamic effects on the structure, minimally impacting the scan consistency on some elements. The inclined façades introduced additional geometric complexity, providing a suitable context to test the methodology. The analysis focused on the column elements, evaluated under criteria C1—C3 of EN 1090-2:2020, and the results are synthesised in a stable vertical behaviour across the structure. All elements stayed under the Class 1 tolerance, except P.53, which presented a 3.7 cm deviation along the Y direction. This deviation likely resulted from incomplete lateral bracing at the time of the survey. In general, the measured displacements showed better performance compared with Case I, see Table 6.

**Figure 14:**
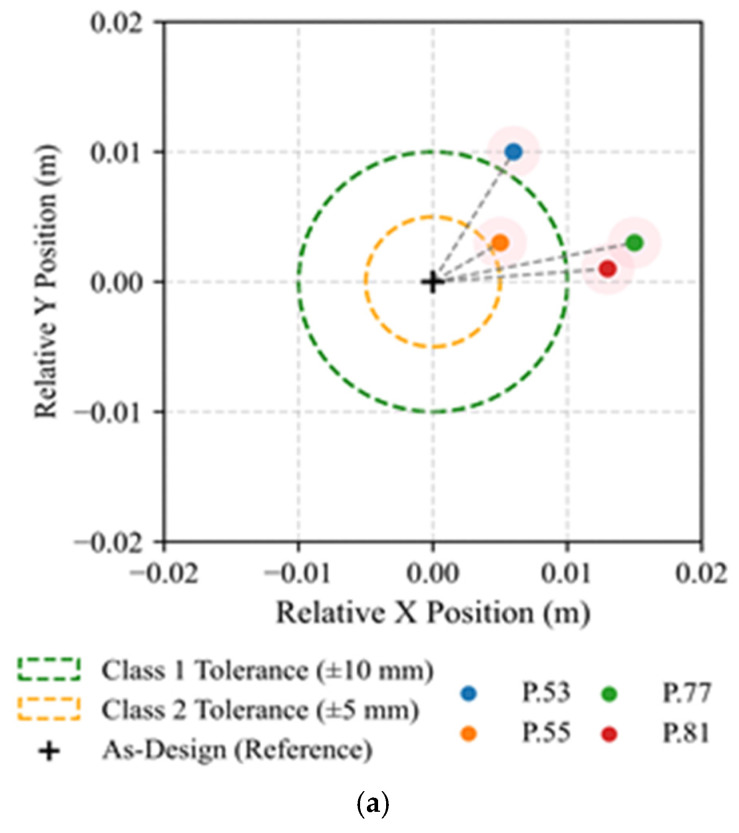

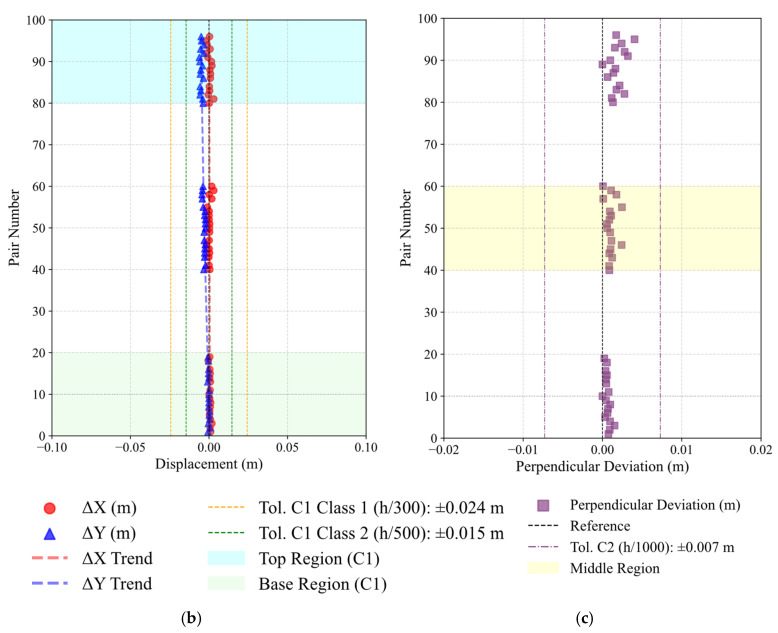
Visual representation of geometric deviation assessment for Case II–Maia: (**a**) Criterion C1—Planimetric positioning of columns, (**b**) Criterion C2—column inclination, (**c**) Criterion C3—column straightness.

Under Criterion C1, the methodology demonstrated consistent performance, with most elements remaining within the tolerance. Columns P.77 and P.81 exhibited marginal horizontal shifts following the global direction of the façade. Nevertheless, the results confirm that direct georeferencing improved positional accuracy and reduced dispersion, as shown in Figure 14a.

The assessment of column inclination revealed a stable vertical behaviour across the structure. All elements stayed under the Class 1 tolerance, except P.53, which presented a 3.7 cm deviation along the Y direction. This deviation likely resulted from incomplete lateral bracing at the time of the survey. In general, the measured displacements showed better performance compared with Case I. The analysis of straightness confirmed negligible deformation along the column axes. All measured mid-region deviation (Δm) remained well below the tolerance limit. Consistent with Case I, these results demonstrate fabrication accuracy and the methodology’s sensitivity to millimetric variations. Figure 14b,c presents two representative examples for both criteria.

## 4. Conclusions

The integration of point cloud data and DL algorithms into construction workflows represents a major advancement in geometric quality control, particularly in automating normative compliance checks. This study presents a novel framework for the automated verification of geometric conformity in accordance with EN 1090-2:2020, combining laser scanning and AI-driven object detection through the YOLOv8 algorithm for steel cross-section analysis. The methodology was tested in two real-world case studies at different assembly stages using two laser scanning systems. Both proved suitable, with scanner positioning emerging as a key factor in capturing accurate element geometry.

The YOLOv8 model was trained on more than 48,501 synthetic and real images, supported by 3D data augmentation techniques and a carefully structured annotation process. Unlike traditional methods, this approach enables comprehensive geometric segmentation while compensating for missing or occluded data. Although the current version of the model is limited to three steel profile series with 100 subclasses, it generalises well across varied acquisition conditions. In Case I, the methodology successfully assessed the geometric displacements of 77% of the captured elements. The lower rate was due to limited accessibility and partition walls that restricted full geometry acquisition. In contrast, Case II achieved a higher assessment rate, with 94% of the columns successfully evaluated. Detected deviations verified conformity with five normative rules. On average, three outliers per element (97% valid data) arose, mainly from overlapping connections affecting model inference.

The model’s performance metrics confirm its robustness. The two-stage training strategy—initially synthetic, then fine-tuned with real images—proved effective for accurate, generalisable predictions. The final model achieved a precision of 94.47% and a recall of 95.84% for both the bounding box and segmentation mask outputs. In terms of segmentation performance, the model reached a mAP@50 of 98.45% and a mAP@50-95 of 70.20%. These results validate the synthetic dataset and 3D augmentation as a solid foundation, with real images being crucial for performance on noisy or occluded data.

While the alignment between TLS-derived point clouds and digital models typically benefits from the use of GCPs, this methodology demonstrated robust performance even in the absence of such a reference system. This reinforces the flexibility and practical applicability of the approach in real-world construction environments.

With an expanded dataset, the framework can be extended to a wider range of steel profiles and other structural materials through dataset expansion and refined annotation. Its flexibility is relevant for both heritage rehabilitation, such as timber framing, and modern Light Steel Framing (LSF) systems, although the applicable standards and tolerance criteria would differ from those defined in EN 1090-2:2020. While this study focused on validating five geometric conformity criteria from EN 1090-2:2020, the framework can integrate additional tolerance criteria for both assembly and fabrication phases. It also supports consistent identification and placement of structural profiles, reducing systematic errors and preserving design intent.

From a practical engineering perspective, the main contribution of this study is the establishment of an end-to-end digital pipeline that replaces conventional manual geometric inspection with an automated, repeatable and auditable process. The proposed Scan-2D Projection-YOLOv8-Validation workflow integrates terrestrial laser scanning, 2D cross-sectional projection, deep learning-based profile identification and automated validation against EN 1090-2:2020. This enables objective and regulation-oriented geometric quality control during steel structure assembly. This approach has been demonstrated to reduce reliance on subjective visual inspection and manual measurements. Furthermore, it has been shown to enhance inspection efficiency and safety. It also provides traceable evidence of geometric conformity, which is suitable for integration into contemporary digital construction workflows.

Future developments may involve the integration of UAV-mounted LiDAR (ALS) to enhance geometric inspection of elevated or inaccessible components. The methodology could also evolve into a real-time tool for on-site verification tools, particularly with augmented reality (AR) systems, enabling operators to visualise geometric displacements during assembly. Additionally, coupling the framework with BIM-based digital twins could enable real-time structural monitoring and dynamic updates to As-Is models based on verified deviations.

## Figures and Tables

**Table 1:** Main technical specification of the equipment used, as reported by [51].

Specification	Leica BLK360 G1	Leica RTC360
Nominal ranging accuracy	±4 mm at 10 m	±1 mm at 10 m
Maximum effective range	60 m	130 m
Measurement rate	360,000 pts/s	2,000,000 pts/s
Field of view	360° × 300°	360° × 300°
Inertial/navigation sensors	IMU	IMU + VIS
Typical scan time (medium density)	3–5 min	<2 min
Weight	1 kg	5.35 kg

**Table 2:** Overview of the YOLOv8 training hyperparameter configuration.

Parameter	Definition
Device	NVIDIA GeForce RTX 4090
Dataset split	70% train/20% val/10% test
Image count	44,167 train/2561 validation/1773 test
Input resolution	640 × 640 px 100 DPI
Batch size	9
Epochs	150
Optimizer	AdamW
Mixed Precision AMP	True
Initial learning rate	9.28 × 10^−5^
Momentum	0.9179
Weight Decay	5.15 × 10^−5^

**Table 3:** Validation metrics of the final YOLOv8 segmentation model.

Metrics	Bounding Box (B)	Masks (M)
Precision (P)	0.9447	0.9447
Recall (R)	0.9584	0.9584
mAP@50	0.9910	0.9845
mAP@50-95	0.9625	0.7020

**Table 4:** Geometric deviation limits defined in EN 1090-2:2020 for assembly of structural steel elements.

Criterion	Visualisation	Description	Parameter	Class 1
C1—Planimetric positioning of columns relative to a reference point	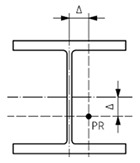	Horizontal offset of the column base centroid from the design reference point (EN 1090-2:2020, Table B.20-1)	Δ = constant value	±1 cm
C2—Column inclination in single-storey structures	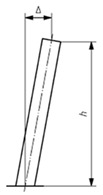	Horizontal displacement between the base and top centroid of the column (EN 1090-2:2020, Table B.17-1)	Δ=±h/300	±*h*/300
C3—Column straightness in single-storey structures	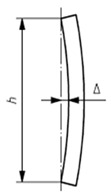	Lateral deviation of the mid-span centroid from the line connecting both ends (EN 1090-2:2020, Table B.17-5)	Δ = ±*h*/1000	±*h*/1000
C4—Beam vertical inclination	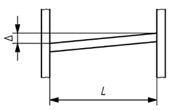	Elevation difference between the two beams ends along its span (EN 1090-2:2020, Table B.15-3)	Δ = ±l/1000	±l/500, ≤1 cm
C5—Beam mid-span straightness in plan	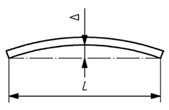	Vertical deviation of the mid-span point from the straight line between extremities (EN 1090-2:2020, Table B.16-3).	Δ = ±l/500	±l/500

**Table 5:** Measured geometric deviations for selected structural elements in Case I according to EN 1090-2:2020.

	Element	P.194	P.200	P.213	P.216	B.95	B.115	B.125	B.133	Class 1 Tolerance [cm]
Criteria		Δx/Δy [cm]	Δx/Δy [cm]	Δx/Δy [cm]	Δx/Δy [cm]	Δx/Δy [cm]	Δx/Δy [cm]	Δx/Δy [cm]	Δx/Δy [cm]	
C1		1.6/0.4	1.8/1.9	0.8/0.6	0.6/0.5	-	-	-	-	±1.0
C2		4.2/1.1	4.4/5.4	0.1/5.4	0.2/5.0	-	-	-	-	±2.4
C3		0.1 ^1^	0.1 ^1^	0.1 ^1^	0.2 ^1^	-	-	-	-	±0.7
C4		-	-	-	-	0.2 ^2^	0.4 ^2^	−1.2 ^2^	−2.2 ^2^	±1.0
C5		-	-	-	-	1.6 ^1^	0.1 ^1^	0.2 ^1^	0.2 ^1^	±1.5

^1^ Δm, maximum perpendicular deviation along the element mid-region; ^2^ Δy, vertical deviation.

**Table 6:** Measured geometric deviations for selected structural elements in Case II according to EN 1090-2:2020.

	Element	P.53	P.55	P.77	P.81	Class 1 Tolerance [cm]
Criteria		Δx/Δy [cm]	Δx/Δy [cm]	Δx/Δy [cm]	Δx/Δy [cm]	
C1		0.6/1.0	0.5/0.3	1.5/0.3	1.3/0.1	±1.0
C2		1.4/3.7	1.2/−1.5	0.2/−0.4	1.8/2.3	±2.4
C3		0.1 ^1^	≈0.0 ^1^	≈0.0 ^1^	0.1 ^1^	±0.7

^1^ Δm, maximum perpendicular deviation along the element mid-region.

## Data Availability

Code and datasets may be available upon request.
